# Derivation and Exploratory Evaluation of the Respiratory Rate-Oxygenation (ROX) Index as a Predictor of High-Flow Nasal Cannula Outcomes in Patients With COVID-19 Pneumonia

**DOI:** 10.7759/cureus.114739

**Published:** 2026-08-18

**Authors:** Rajesh Chandra, Ankita, Harish Kumar, Sandeep Kumar, Ashutosh Kaushal, Anuj Jain, Nitesh Sinha

**Affiliations:** 1 Anaesthesiology, Rajendra Institute of Medical Sciences, Ranchi, IND; 2 Trauma and Emergency Medicine, All India Institute of Medical Sciences, Bhopal, Bhopal, IND; 3 Anaesthesiology, All India Institute of Medical Sciences, Bhopal, Bhopal, IND

**Keywords:** acute hypoxemic respiratory failure, covid-19, high-flow nasal cannula, mechanical ventilation, non-invasive ventilation, rox index

## Abstract

Objectives: High-flow nasal cannula (HFNC) therapy is a first-line respiratory support modality for acute hypoxemic respiratory failure (AHRF) due to COVID-19 pneumonia. The respiratory rate-oxygenation (ROX) index, defined as the ratio of peripheral oxygen saturation (SpO_2_)/fraction of inspired oxygen (FiO_2_) divided by respiratory rate, has been proposed as a predictor of HFNC success or failure. This study aimed to derive time-specific cut-off values and explore the predictive accuracy of the ROX index in COVID-19 patients receiving HFNC therapy.

Methods: This single-center prospective observational cohort study included 60 consecutive adult patients with COVID-19 pneumonia requiring HFNC therapy in the intensive care unit (ICU) from March 2021 to February 2022. The study was conducted at Medanta, Ranchi, India. The ROX index was measured at 2, 6, 12, 18, and 24 hours after HFNC initiation. HFNC failure was defined as the subsequent requirement for invasive mechanical ventilation (IMV) within the index ICU admission. Receiver operating characteristic (ROC) curves with optimal cut-off determined by the Youden index, and univariate logistic regression were used to assess predictive accuracy.

Results: Of 60 patients enrolled, 37/60 (61.7%) achieved HFNC success, and 23/60 (38.3%) experienced HFNC failure. The ROX index was significantly higher in the success group at all measured time points (P<0.001). The ROX index at 12 hours was the best predictor of HFNC outcome (area under the curve (AUC)=0.973, sensitivity 36/37 (97.3%), specificity 19/23 (82.6%), cut-off value 5.81). These performance estimates were derived and evaluated in the same 60-patient cohort and should be considered optimistic pending external validation. Univariate logistic regression showed odds ratios up to 102 at 24 hours. Vaccination was present in 25/60 patients (41.7%) and showed a non-significant trend toward association with HFNC success (chi-square 3.72, P=0.054). Mortality was 18/23 (78.3%) in the failure group versus 0/37 (0%) in the success group (P<0.001).

Conclusions: The ROX index is a candidate non-invasive bedside predictor of HFNC therapy outcome in patients with COVID-19 pneumonia. Derived cut-offs, particularly at 12 hours (5.81), showed promising predictive accuracy and can aid clinicians in timely decisions regarding escalation to IMV. These findings are hypothesis-generating and require external validation before clinical implementation.

## Introduction

The COVID-19 pandemic has presented unprecedented challenges in the management of acute hypoxemic respiratory failure (AHRF), one of the most common complications associated with severe SARS-CoV-2 infection [1,2]. Various non-invasive respiratory support modalities, including high-flow nasal cannula (HFNC) and non-invasive ventilation (NIV), have emerged as effective techniques because they can deliver high fractions of inspired oxygen, improve patient comfort, and reduce the need for invasive mechanical ventilation (IMV) in selected patients [3-5].

Despite these benefits, the major limitation of HFNC is the risk of delayed intubation when therapy fails, which can worsen outcomes [6,7]. The respiratory rate-oxygenation (ROX) index, defined as the ratio of peripheral oxygen saturation (SpO_2_)/fraction of inspired oxygen (FiO_2_) divided by respiratory rate, has been validated in non-COVID pneumonia as a predictor of HFNC success or failure [8,9]. However, COVID-19 pneumonia has unique clinical features, including silent hypoxemia and altered ventilatory drive, which may affect the relationship between oxygenation, respiratory rate, and clinical deterioration [1,2].

This study prospectively aimed to (1) derive time-specific optimal cut-off values for the ROX index in a prospective cohort of COVID-19 patients receiving HFNC therapy; and (2) explore the association of the ROX index with ICU outcomes, including mortality and serial Sequential Organ Failure Assessment (SOFA) score trends [10-12]. The primary outcome was HFNC failure (defined as requirement for IMV); secondary outcomes were ICU length of stay, mortality, and SOFA score trends over the first five days.

## Materials and methods

Study design and setting

This was a single-center prospective observational cohort study conducted over one year, from March 2021 to February 2022, in three intensive care units (ICUs) at Medanta, Ranchi, India. The study was approved by the institutional ethics committee of Medanta, Ranchi (approval number: MR/ADMIN/2021/0072). Written informed consent was obtained from all participants or their legally authorized representatives.

**Figure 1:**
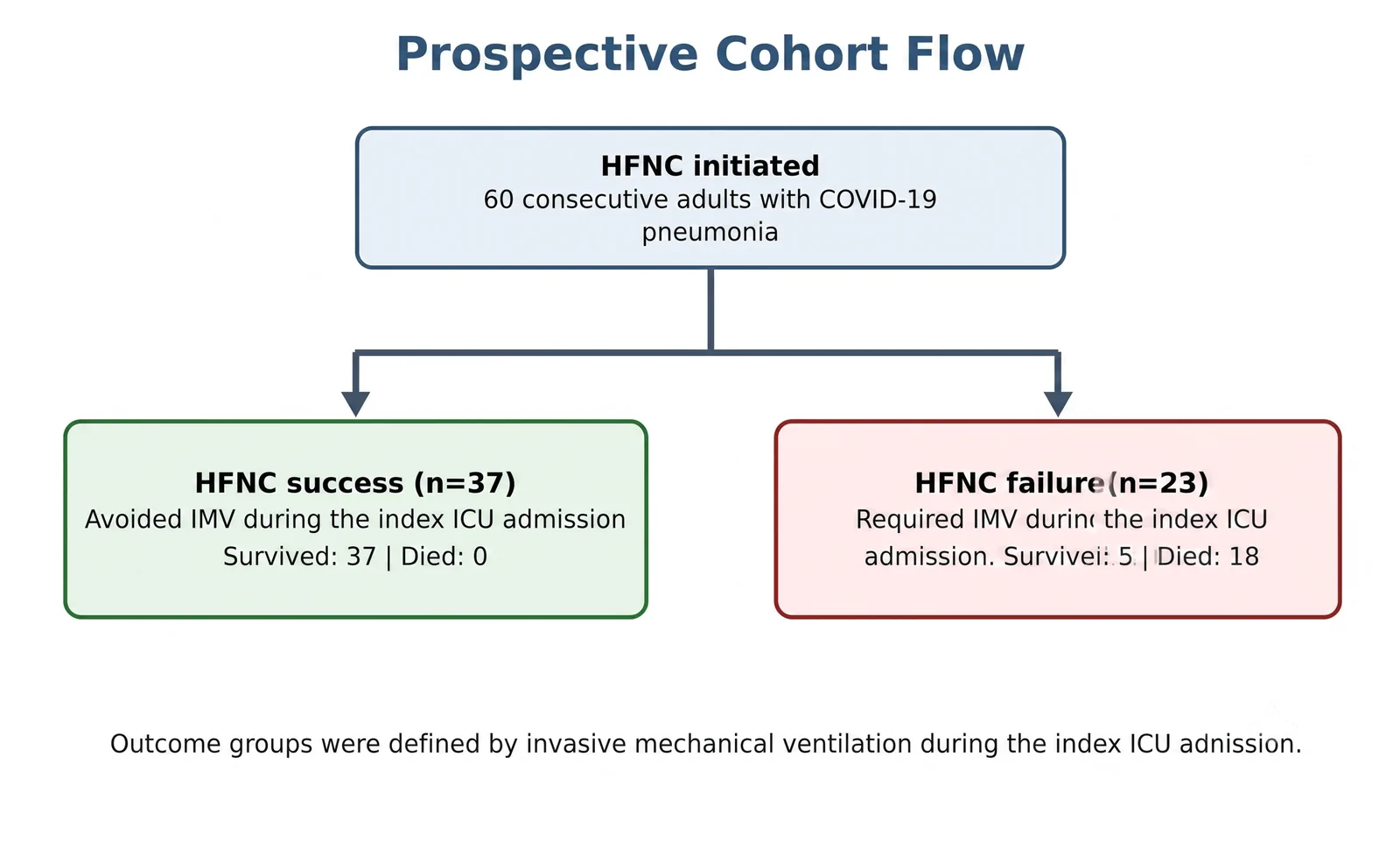
Participant flow and observed outcomes Sixty consecutive adults initiated high-flow nasal cannula (HFNC) therapy; 37 avoided invasive mechanical ventilation (IMV), whereas 23 required IMV during the index intensive care unit (ICU) admission. Among patients who experienced HFNC failure, five survived, and 18 died.

The study enrollment, allocation, follow-up, and analysis are summarized in Figure 1, which presents a Consolidated Standards of Reporting Trials (CONSORT)-style flow diagram.

Study population

The inclusion criteria were adult patients aged ≥18 years with confirmed COVID-19 pneumonia by reverse transcription polymerase chain reaction (RT-PCR), peripheral oxygen saturation (SpO_2_) <94% while receiving conventional oxygen therapy, and a requirement for HFNC therapy.

The exclusion criteria were age <18 years, non-COVID-19 AHRF, prior IMV, do-not-resuscitate/do-not-intubate orders, pregnancy, an immediate indication for intubation at presentation, and elective intubation for diagnostic or therapeutic procedures.

Sample size calculation

The sample size was calculated to estimate the sensitivity of the ROX index for prediction of HFNC failure. Assuming an expected sensitivity of 90%, absolute precision of 10%, and a 95% confidence level, the required number of HFNC-failure cases was 35 (formula: $n = \frac{Z^2 \times \mathrm{sensitivity} \times (1-\mathrm{sensitivity})}{d^2} = \frac{1.96^2 \times 0.90 \times 0.10}{0.10^2} = 34.6$, rounded to 35). Based on an anticipated HFNC failure proportion of approximately 38%, the required total sample size was 35/0.38 ≈ 92. However, based on a success proportion of approximately 60%, the total sample was 35/0.60 ≈ 58. Therefore, 60 consecutive patients were enrolled to maintain adequate precision and account for incomplete observations.

HFNC protocol and ROX index measurement

HFNC therapy was initiated with flow rates of 30-60 L/min and FiO_2_ of 40-100%, titrated to maintain SpO_2_ between 92% and 96%. HFNC therapy was delivered using the Optiflow+ HFNC system (Fisher & Paykel Healthcare, Auckland, New Zealand). FiO_2_ was the set device value, recognizing that actual delivered FiO_2_ may vary with flow rate and cannula fit. SpO_2_ was recorded as the displayed value; values >97% were noted as potentially invalidating the SpO_2_/FiO_2_ ratio. Respiratory rate was counted manually over 60 seconds by the attending nurse or physician at each time point; assessors were not blinded to group outcome. The ROX index was calculated as (SpO_2_/FiO_2_)/respiratory rate and measured at 2, 6, 12, 18, and 24 hours after HFNC initiation. HFNC failure was defined as the subsequent requirement for IMV within the index ICU admission.

HFNC failure and escalation criteria

For patients who required intubation before a scheduled measurement time point, the ROX index was not measured at that time point and no forward carry of values was applied. The time-to-intubation distribution (median, IQR) and number of patients intubated before 12 and 24 hours are reported in the Results.

Co-interventions

Co-interventions included awake prone positioning, corticosteroids (dexamethasone), tocilizumab, remdesivir, anticoagulation (prophylactic/therapeutic), and a trial of NIV, including continuous positive airway pressure (CPAP) or bilevel positive airway pressure (BiPAP). NIV (CPAP or BiPAP) was used prior to tracheal intubation and IMV in patients who failed HFNC.

SOFA score calculation

The SOFA score was calculated using the original method described by Vincent et al. [12]. The score evaluates six organ systems: respiratory, coagulation, liver, cardiovascular, central nervous system, and renal. Each organ system is scored 0-4 according to the worst physiological or laboratory value during the preceding 24 hours. Daily SOFA scores were recorded during the first five days of HFNC therapy. For patients who died or were intubated before day 5, SOFA scores were recorded only up to the day of the event; no imputation was performed.

Outcome measures

The primary outcome was HFNC failure, defined as the requirement for IMV within the index ICU admission. HFNC success was defined as avoidance of IMV. Secondary outcomes included hypercapnia, defined as a partial pressure of arterial carbon dioxide (PaCO_2_) >45 mmHg or a partial pressure of venous carbon dioxide (PvCO_2_) >50 mmHg, where measured; ICU length of stay; 28-day mortality; and trends in SOFA scores over the first five days.

Statistical analysis

Data were analyzed using IBM SPSS Statistics for Windows, Version 23 (Released 2016; IBM Corp., Armonk, New York, United States). Continuous variables are presented as mean ± SD and compared using Welch's independent-samples t-test. Categorical variables are presented as frequencies/percentages and compared using the Pearson's chi-square test or Fisher's exact test (expected cell count <5). Receiver operating characteristic (ROC) curves were constructed to determine optimal ROX index cut-off values at each time point; the optimal cut-off was selected by maximizing the Youden index (sensitivity + specificity - 1). Area under the curve (AUC) confidence intervals (CIs) were calculated using the DeLong method. Sensitivity, specificity, positive predictive value (PPV), negative predictive value (NPV), AUC, and 95% CIs were calculated. Univariate logistic regression was used to calculate ORs, 95% CIs, Wald chi-square statistics, and P-values. P<0.05 was considered statistically significant. No correction was applied for testing across five time points; all performance estimates from this derivation cohort should be interpreted as potentially optimistic.

This study was conducted and reported in accordance with the Strengthening the Reporting of Observational Studies in Epidemiology (STROBE) guidelines.

## Results

Baseline characteristics

A total of 60 patients were enrolled: 37/60 (61.7%) avoided IMV and 23/60 (38.3%) required IMV during the index ICU admission. Baseline characteristics are presented in Table 1.

**Table 1:** Baseline demographic and clinical characteristics (N=60) Welch's independent-samples t-test was used for continuous variables; Pearson's chi-square or Fisher's exact test was used for categorical variables. NS: not statistically significant; SOFA: Sequential Organ Failure Assessment; HFNC: high-flow nasal cannula; SD: standard deviation

Characteristic	HFNC success (n=37)	HFNC failure (n=23)	Test statistic	P-value
Age (years), mean±SD	51.8±11.2	53.1±11.8	t=-0.43	0.65
Male sex, n (%)	24 (64.9)	14 (60.9)	chi-square=0.10	0.75
Diabetes mellitus, n (%)	12 (32.4)	9 (39.1)	chi-square=0.28	0.59
Hypertension, n (%)	10 (27.0)	7 (30.4)	chi-square=0.08	0.77
Chronic kidney disease, n (%)	4 (10.8)	3 (13.0)	Fisher exact	0.79
Coronary artery disease, n (%)	3 (8.1)	2 (8.7)	Fisher exact	0.93
Vaccinated, n (%)	19 (51.4)	6 (26.1)	chi-square=3.72	0.054 (NS)
Baseline SOFA score, mean±SD	15.11±1.52	15.04±1.26	t=0.19	0.85

Comorbidities, including diabetes mellitus (35%), hypertension (28.3%), chronic kidney disease (11.7%), and coronary artery disease (8.3%), are detailed in Table 2.

**Table 2:** Comorbidities distribution (N=60) Percentages are calculated using the total study population (N=60).

Comorbidity	n (%)
Diabetes mellitus	21 (35.0)
Hypertension	17 (28.3)
Chronic kidney disease	7 (11.7)
Coronary artery disease	5 (8.3)
None	10 (16.7)

Twenty-five patients (41.7%) had received COVID-19 vaccination; the relationship between vaccination status and HFNC outcome is shown in Table 3. Vaccination status showed a non-significant trend toward association with HFNC success (chi-square 3.72, P=0.054). Baseline SOFA scores were similar between the success and failure groups.

**Table 3:** Vaccination status and HFNC outcome (N=60) HFNC: high-flow nasal cannula; Statistical test: Pearson's chi-square

Vaccination status	HFNC success n=37, n (%)	HFNC failure n=23, n (%)	Total N=60, n (%)	Test; P-value
Vaccinated	19 (51.4)	6 (26.1)	25 (41.7)	Pearson chi-square=3.72; P=0.054
Unvaccinated	18 (48.6)	17 (73.9)	35 (58.3)	-

ROX index values

ROX values were higher in the HFNC-success group than in the HFNC-failure group at all reported time points (P<0.001; Table 4). In the success group, mean ROX index values were 5.64±0.90 at two hours, 5.06±0.92 at six hours, 4.84±0.05 at 12 hours, 4.77±0.91 at 18 hours, and 6.31±0.46 at 24 hours. In the failure group, mean ROX index values were 6.96±0.79 at two hours, 7.08±0.85 at six hours, 7.42±1.01 at 12 hours, 7.20±1.10 at 18 hours, and 7.73±1.15 at 24 hours (Table 4).

**Table 4:** ROX index values at different time points (mean±SD) HFNC: high-flow nasal cannula; ROX: ratio of oxygen saturation to fraction of inspired oxygen to respiratory rate; SD: standard deviation; Statistical test: Welch's independent-samples t-test

Time point	HFNC success (n=37)	HFNC failure (n=23)	Test statistic	P-value
2 hours	5.64±0.90	6.96±0.79	F=65.78	<0.001
6 hours	5.06±0.92	7.08±0.85	F=68.51	<0.001
12 hours	4.84±0.05	7.42±1.01	F=15.51	<0.001
18 hours	4.77±0.91	7.20±1.10	t=9.27	<0.001
24 hours	6.31±0.46	7.73±1.15	F=6.70	<0.001

Predictive accuracy of the ROX index

ROC curve analysis demonstrated that the ROX index at 12 hours had the highest AUC (0.973), with an optimal cut-off of 5.81 (Youden index), sensitivity 97.3%, specificity 82.6%, PPV 90.0%, and NPV 95.0% (Table 5). Note that PPV and NPV are prevalence-dependent and reflect this cohort's 61.7% HFNC success rate.

**Table 5:** Predictive accuracy of ROX index for HFNC success AUC: area under the receiver operating characteristic (ROC) curve; CI: confidence interval by the DeLong method; NPV: negative predictive value; PPV: positive predictive value; HFNC: high-flow nasal cannula; Optimal cut-off by Youden's index

Time	Cut-off	Sensitivity (%)	Specificity (%)	PPV (%)	NPV (%)	AUC	95% CI	χ² (P)
2 hours	5.12	97.3	65.2	81.8	93.7	0.87	0.78-0.96	11.44 (0.001)
6 hours	5.87	94.6	65.2	81.4	88.2	0.92	0.86-0.98	24.98 (0.001)
12 hours	5.81	97.3	82.6	90.0	95.0	0.973	0.94-1.00	40.75 (0.001)
18 hours	4.79	97.3	56.5	78.3	92.9	0.964	0.92-1.00	15.75 (0.001)
24 hours	6.00	97.3	73.9	85.7	94.4	0.845	0.75-0.94	7.52 (0.006)

**Figure 2:**
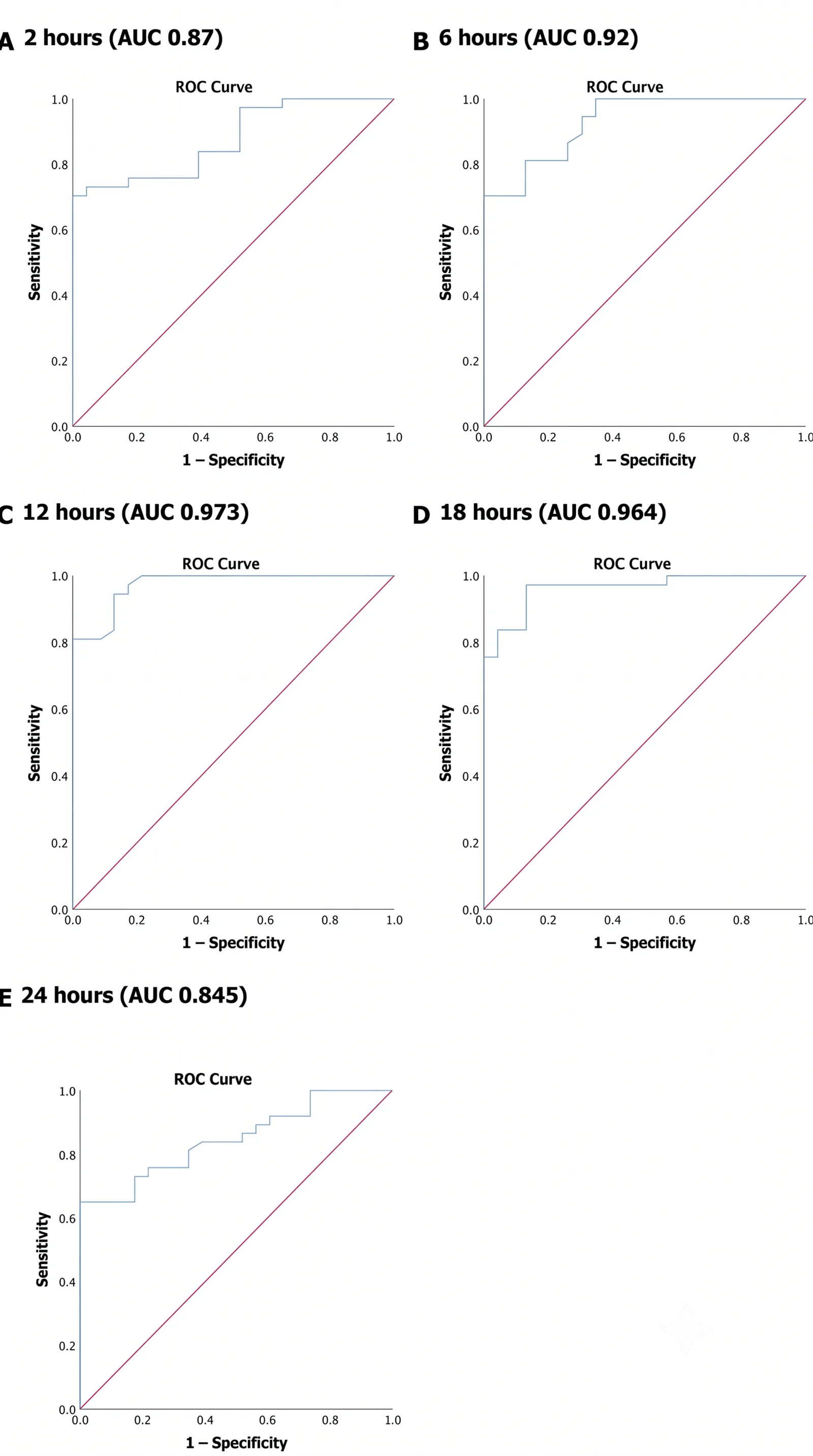
Receiver operating characteristic (ROC) curves for the ROX index for prediction of HFNC success at five time points after HFNC initiation (A) ROX index at two hours (AUC 0.87); (B) ROX index at six hours (AUC 0.92); (C) ROX index at 12 hours (AUC 0.973); (D) ROX index at 18 hours (AUC 0.964); and (E) ROX index at 24 hours (AUC 0.845). In each panel, the blue line represents the ROC curve for the corresponding time point, and the red diagonal line represents the reference line of no discrimination. ROC curves correspond to the AUC values and time-specific cut-off values summarized in Table 5. AUC: area under the curve; HFNC: high-flow nasal cannula; ROX: ratio of oxygen saturation to fraction of inspired oxygen to respiratory rate

The ROC curves corresponding to Table 5 are provided in Figure 2.

Univariate logistic regression

Univariate logistic regression analysis showed strong associations between the ROX index and HFNC success at all time points (Table 6). The OR was 67.50 (95% CI 7.75-587.88, P=0.001) at two hours, 32.82 (95% CI 6.21-173.15, P<0.001) at six hours, 83.12 (95% CI 13.92-496.35, P<0.001) at 12 hours, 46.80 (95% CI 5.44-402.28, P=0.0005) at 18 hours, and 102.00 (95% CI 11.36-915.23, P<0.001) at 24 hours.

**Table 6:** Univariate binary logistic regression analysis of time-specific ROX index cut-offs for prediction of HFNC success (N=60) Binary outcome used in all models: HFNC success=1 and HFNC failure requiring invasive mechanical ventilation=0. Each predictor is dichotomized at the corresponding time-specific optimal ROX index cut-off shown in Table 5. CI: confidence interval; HFNC: high-flow nasal cannula; ROX: ratio of oxygen saturation to fraction of inspired oxygen to respiratory rate; * Statistically significant (P<0.05)

Predictor variable	Binary outcome	Odds ratio	95% CI	Regression test statistic	P-value
ROX >5.12 at 2 hours	HFNC success=1; failure=0	67.50	7.75-587.88	Wald chi-square=14.55	0.001*
ROX >5.87 at 6 hours	HFNC success=1; failure=0	32.82	6.21-173.15	Wald chi-square=16.91	<0.001*
ROX >5.81 at 12 hours	HFNC success=1; failure=0	83.12	13.92-496.35	Wald chi-square=23.51	<0.001*
ROX >4.79 at 18 hours	HFNC success=1; failure=0	46.80	5.44-402.28	Wald chi-square=12.27	0.0005*
ROX >6.00 at 24 hours	HFNC success=1; failure=0	102.00	11.36-915.23	Wald chi-square=17.06	<0.001*

SOFA score trends

SOFA scores were similar between the success and failure groups on day 1 but diverged progressively by days 3-5. The temporal trends in SOFA scores for both groups over five days are illustrated in Table 7.

**Table 7:** SOFA score trends over five days Values are mean±SD. Welch's independent-samples t-test was used. *Statistically significant (P<0.05). HFNC: high-flow nasal cannula; SOFA: Sequential Organ Failure Assessment

Time point	HFNC success (n=37)	HFNC failure (n=23)	Test statistic	P-value
Day 1	15.11±1.52	15.04±1.26	t=0.19	0.85
Day 2	12.86±1.87	15.81±1.43	t=-6.89	<0.001*
Day 3	10.24±2.14	16.94±1.67	t=-13.53	<0.001*
Day 4	7.83±2.33	17.82±1.82	t=-18.53	<0.001*
Day 5	5.21±2.44	18.73±2.01	t=-23.31	<0.001*

All 37/37 (100%) patients in the HFNC success group survived without IMV. In the HFNC failure group, 5/23 (21.7%) survived after escalation to IMV and 18/23 (78.3%) died. The difference in mortality between groups was statistically significant (Table 8).

**Table 8:** Clinical outcomes according to HFNC outcome group Pearson's chi-square test was used for mortality comparison. Statistically significant (P<0.05). HFNC: high-flow nasal cannula

Clinical outcome	HFNC success (n=37)	HFNC failure (n=23)	Test statistic	P-value
HFNC failure requiring invasive mechanical ventilation with survival	0 (0)	5 (21.7)	-	-
Death	0 (0)	18 (78.3)	37.72(Yates)	<0.001

## Discussion

This prospective observational study provides derivation-level evidence for the ROX index as a bedside predictor of HFNC therapy outcome in adults with COVID-19 pneumonia. The principal finding was that the 12-hour ROX index had the highest predictive accuracy within this cohort, with an AUC of 0.973 and an optimal cut-off value of 5.81. Because the cut-offs were derived and evaluated in the same 60 patients, these AUC values reflect internal (optimistically biased) performance estimates. External validation in an independent cohort is required before these thresholds can be recommended for clinical use. These findings support serial ROX monitoring during the first 24 hours of HFNC therapy rather than reliance on a single baseline measurement.

Our findings are biologically and clinically consistent with the original ROX studies by Roca et al., in which higher ROX values after HFNC initiation identified patients at lower risk of intubation in pneumonia-related hypoxemic respiratory failure [8,9]. The present 12-hour cut-off of 5.81 is higher than the classical non-COVID threshold of 4.88, which may reflect differences in COVID-19 physiology, including silent hypoxemia and altered ventilatory drive. However, local FiO_2_ titration practice, intubation thresholds, and case mix are at least equally plausible alternative explanations that cannot be excluded in a single-center study.

In COVID-19 cohorts, Chandel et al. reported that ROX trends were useful for predicting HFNC success and emphasized the adverse implications of delayed HFNC failure [10]. Panadero et al. also found that HFNC was useful in patients with COVID-19-associated acute respiratory distress syndrome (ARDS) and reported that lower ROX values predicted intubation [11].

Ferrer et al. identified a 24-hour ROX cut-off of 5.35 for predicting HFNC success in acute respiratory failure due to SARS-CoV-2, whereas Valencia et al. reported moderate discriminatory performance of the ROX index compared with the heart rate, acidosis, consciousness, oxygenation, and respiratory rate (HACOR) score in patients with SARS-CoV-2 pneumonia [13,14]. In this context, the high 12-hour AUC observed in our cohort suggests strong discrimination within this sample, although it should be interpreted cautiously because of the derivation design, single-center setting, and modest sample size.

The mortality difference between HFNC success and failure groups was large, with 18/23 (78.3%) deaths in the failure group and no deaths in the success group. This finding is aligned with earlier reports that delayed intubation after HFNC failure is associated with worse outcomes [6]. However, because HFNC success is defined as avoidance of IMV, the 0% versus 78.3% mortality comparison is partly definitional and should not be interpreted as evidence that ROX-guided management improves survival. No data on time from HFNC initiation to intubation are presented, which is the variable that would be needed to support the delayed-intubation argument.

The study also showed that vaccination status had a non-significant trend toward association with HFNC success (chi-square 3.72, P=0.054). This finding does not meet conventional thresholds for statistical significance and should be interpreted cautiously given the small number of vaccinated patients (n=25), absence of vaccine type/dose information, and lack of multivariable adjustment. The observational design, sample size, and lack of multivariable adjustment limit causal interpretation.

Limitations

This study has several limitations. First, it was conducted at a single center with 60 patients, which limits generalizability. Second, only univariate analysis was performed; multivariable analysis would be preferable for adjustment of confounders. Third, the study was conducted between March 2021 and February 2022; findings may not apply equally across different SARS-CoV-2 variants or vaccination coverage levels. Fourth, the relatively high AUC values should be externally validated in larger multicenter cohorts. Fifth, cut-off values were derived and evaluated in the same 60-patient cohort, with five time points tested without multiplicity adjustment or internal validation (bootstrap or cross-validation optimism correction). The reported AUCs should therefore be considered optimistic estimates. Sixth, handling of missing ROX values for patients intubated before a given time point is not explicitly described, and the time-to-intubation distribution is not reported. Seventh, co-interventions (prone positioning, corticosteroids, tocilizumab, remdesivir, anticoagulation) are not reported and may be major determinants of HFNC outcome.

Strengths

The strengths of this study include its prospective design, consecutive enrollment, clinically relevant definition of HFNC failure, and serial ROX assessment at multiple time points during the first 24 hours of therapy. The addition of ROC curves, time-specific cut-offs, classification statistics, and logistic regression test statistics improves transparency and interpretability. A complete CONSORT-style flow diagram (Figure 1) documents the study population.

Clinical implications

The ROX index is simple, non-invasive, repeatable, and feasible in resource-limited settings. Based on these exploratory findings, a ROX index <5.81 at 12 hours may be considered a hypothesis-generating threshold that warrants further evaluation. Until external validation is available, this cut-off should not be used as a standalone trigger for escalation decisions.

Future directions

Future studies should externally validate the derived cut-offs, particularly the 12-hour threshold, in larger multicenter cohorts and should evaluate whether ROX-guided escalation protocols reduce delayed intubation, mortality, ICU length of stay, and ventilator-free days. Formal comparison of time-specific AUCs using DeLong testing would clarify whether the 12-hour time point truly outperforms adjacent time points. Additional models incorporating heart rate, vasopressor requirement, biomarkers, and dynamic SOFA trends may further improve predictive performance [15-17].

## Conclusions

The ROX index is a candidate non-invasive bedside predictor of HFNC therapy outcome in patients with COVID-19 pneumonia. Derived cut-offs, particularly at 12 hours (ROX <5.81), showed promising discriminatory accuracy within this single-center cohort. These findings are hypothesis-generating and require external validation in larger, independent cohorts before implementation in clinical practice guidelines.

## References

[1] Grasselli G, Zangrillo A, Zanella A (2020). Baseline characteristics and outcomes of 1591 patients infected with SARS-CoV-2 admitted to ICUs of the Lombardy region, Italy. JAMA.

[2] Guan WJ, Ni ZY, Hu Y (2020). Clinical characteristics of coronavirus disease 2019 in China. N Engl J Med.

[3] Rochwerg B, Einav S, Chaudhuri D (2020). The role for high flow nasal cannula as a respiratory support strategy in adults: a clinical practice guideline. Intensive Care Med.

[4] Frat JP, Thille AW, Mercat A (2015). High-flow oxygen through nasal cannula in acute hypoxemic respiratory failure. N Engl J Med.

[5] Ni YN, Luo J, Yu H, Liu D, Liang BM, Liang ZA (2018). The effect of high-flow nasal cannula in reducing the mortality and the rate of endotracheal intubation when used before mechanical ventilation compared with conventional oxygen therapy and noninvasive positive pressure ventilation. A systematic review and meta-analysis. Am J Emerg Med.

[6] Kang BJ, Koh Y, Lim CM (2015). Failure of high-flow nasal cannula therapy may delay intubation and increase mortality. Intensive Care Med.

[7] Demoule A, Vieillard Baron A, Darmon M (2020). High-flow nasal cannula in critically ill patients with severe COVID-19. Am J Respir Crit Care Med.

[8] Roca O, Messika J, Caralt B, García-de-Acilu M, Sztrymf B, Ricard JD, Masclans JR (2016). Predicting success of high-flow nasal cannula in pneumonia patients with hypoxemic respiratory failure: the utility of the ROX index. J Crit Care.

[9] Roca O, Caralt B, Messika J (2019). An index combining respiratory rate and oxygenation to predict outcome of nasal high-flow therapy. Am J Respir Crit Care Med.

[10] Chandel A, Patolia S, Brown AW (2021). High-flow nasal cannula therapy in COVID- 19: using the ROX index to predict success. Respir Care.

[11] Panadero C, Abad-Fernández A, Rio-Ramirez MT (2020). High-flow nasal cannula for acute respiratory distress syndrome (ARDS) due to COVID-19. Multidiscip Respir Med.

[12] Vincent JL, Moreno R, Takala J (1996). The SOFA (Sepsis-related Organ Failure Assessment) score to describe organ dysfunction/failure. On behalf of the Working Group on Sepsis-Related Problems of the European Society of Intensive Care Medicine. Intensive Care Med.

[13] Valencia CF, Lucero OD, Castro OC, Sanko AA, Olejua PA (2021). Comparison of ROX and HACOR scales to predict high-flow nasal cannula failure in patients with SARS-CoV-2 pneumonia. Sci Rep.

[14] Ferrer S, Sancho J, Bocigas I (2021). ROX index as predictor of high flow nasal cannula therapy success in acute respiratory failure due to SARS-CoV-2. Respir Med.

[15] Prakash J, Bhattacharya PK, Yadav AK, Kumar A, Tudu LC, Prasad K (2021). ROX index as a good predictor of high flow nasal cannula failure in COVID-19 patients with acute hypoxemic respiratory failure: a systematic review and meta-analysis. J Crit Care.

[16] Goh KJ, Chai HZ, Ong TH, Sewa DW, Phua GC, Tan QL (2020). Early prediction of high flow nasal cannula therapy outcomes using a modified ROX index incorporating heart rate. J Intensive Care.

[17] Patel M, Chowdhury J, Mills N (2021). Utility of the ROX index in predicting intubation for patients with COVID-19-related hypoxemic respiratory failure receiving high-flow nasal therapy: retrospective cohort study. JMIRx Med.

